# Unilateral Lymphangiomatous Polyp of the Palatine Tonsil in a Very Young Child: A Clinicopathologic Case Report

**DOI:** 10.1155/2011/451542

**Published:** 2012-01-04

**Authors:** Edward Park, Seth M. Pransky, Denise M. Malicki, Paul Hong

**Affiliations:** ^1^Division of Otolaryngology Head and Neck Surgery, Department of Surgery, Dalhousie University, Halifax, Nova Scotia, Canada B3J 3G9; ^2^Division of Pediatric Otolaryngology, University of California-San Diego, San Diego, CA 92123, USA; ^3^Division of Pediatric Pathology, University of California-San Diego, San Diego, CA 92123, USA; ^4^Department of Otolaryngology, IWK Health Centre, 5850/5980 University Avenue, P.O. Box 9700, Halifax, NS, Canada B3K 6R8

## Abstract

Childhood lymphangiomatous polyp of the palatine tonsil is a very unusual lesion found in the head and neck. Tonsillectomy has been reported to be the curative procedure of choice for this lesion. We report a case of a very young child with unilateral lymphangiomatous polyp of the palatine tonsil.

## 1. Introduction

Lymphangioma or lymphatic malformation is a benign congenital tumor of the lymphovascular system that most commonly occurs in the head and neck region [1]. Lymphangiomatous polypoid lesions also arise from the lymphatic and vascular systems but are histologically different than a typical lymphatic malformation [2, 3]. The polypoid masses tend to have varying degrees of fibrous, adipose, and lymphoid components and they tend to occur most commonly in the lower gastrointestinal tracts [4–7].

Lymphangiomatous polypoid lesions of the head and neck are very rare and case reports of these tumors arising from the palatine tonsils in the literature are sparse [3, 8–10].

In this paper, we describe a case of a very young child who was found to have a lymphangiomatous polypoid lesion of the palatine tonsil.

## 2. Case Report

**Figure 1:**
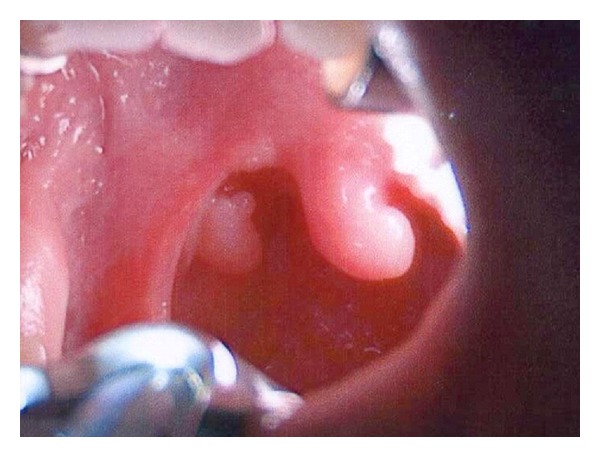
Intraoperative photograph showing a pedunculated polypoid lesion of the right palatine tonsil.

A 3-year-old girl was seen in the pediatric otolaryngology clinic for evaluation of dysphagia to solid foods. She had no other local or systemic symptoms. Esophagogastroduodenoscopy was arranged to rule out eosinophilic esophagitis and any structural pathologies. Intraoperatively, she was found to have a polypoid lesion arising from the superior pole of the right palatine tonsil (Figure 1). Interestingly, the tonsillar lesion was not noted during the clinical exam at the initial consultation visit. After obtaining an additional informed consent with the parents, the lesion was excised for biopsy purposes.

**Figure 2:**
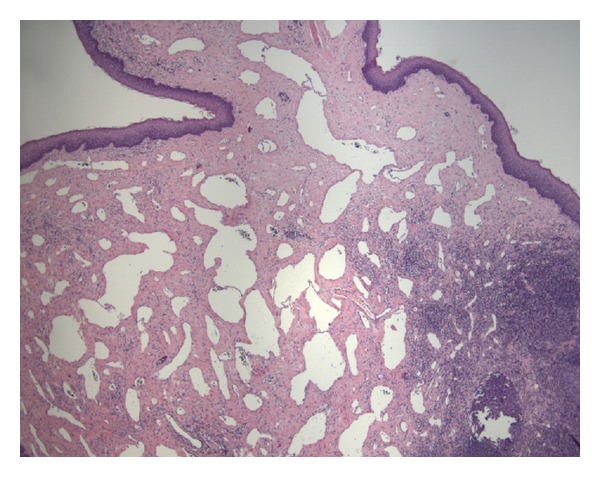
Histopathologic specimen showing proliferation of thin-walled lymphovascular channels containing luminal valve structures and sparse proteinaceous material with fibrous background, surrounding lymphoid tissue, and surface nonkeratinizing stratified squamous epithelium (40x magnification).

The endoscopic examination was normal and eosinophilic esophagitis was ruled out. The histopathologic sections of the lesion arising from the tonsil showed a polypoid portion of fibrous connective tissue with nonkeratinizing stratified squamous epithelium at the surface and dense lymphoid tissue at the base (Figure 2). Central portion of the specimen contained a proliferation of dilated lymphatic channels showing thin epithelial walls, valve structures, and sparse luminal proteinaceous and lymphocyte contents. All of these findings were consistent with a diagnosis of lymphangiomatous polyp of the palatine tonsil. There were no atypical features or evidence of malignancy.

Postoperative followup at one year revealed no evidence of any residual or recurrent polypoid disease. Interestingly, the symptoms of dysphagia gradually improved without any other interventions.

## 3. Discussion

Lymphangiomatous polyps are uncommon benign lesions mostly occurring in the lower gastrointestinal tracts, including the colon [4, 6], the stomach [5, 7], and the small intestine [7]. In the head and neck, palatine tonsil is the most common site of the polyps.

To date, there has been just over 30 cases of tonsillar lymphangiomatous polyps reported in the literature [3, 8–10]. Of these, 26 cases were identified in a single retrospective case series from the Otorhinolaryngic-Head and Neck Tumor Registry of the Armed Forces Institute of Pathology [3].

The incidence of these tumors in the general population is currently unknown but many authors suggest that the true incidence is most likely higher than expected [2, 3]. This is mainly thought to be due to the lack of awareness of the tumor by the clinician and the confusing histologic nomenclature used to describe benign lymphatic lesions. For example, lymphangiomatous polypoid lesions have also been called angiofibromas, fibroangiomas, and angiomas in the pathology literature [3]. In addition to this overlap in terminology, the nature of the tonsil, as a lymphoid organ rich in lymphocytes and efferent lymphatics, suggests that the lymphangiomatous polyps of the tonsil may not be as rare as it would seem and may be underrecognized and underreported [2, 10].

Prior cases of lymphangiomatous polyp of the palatine tonsil have shown that it mostly occurs unilaterally and does not appear to have a gender predilection. The average age of patients in the aforementioned large case series was 25 years [3], and most other articles report a similar adult age at presentation [10]. The patient presented in this paper was 3 years old, which is one of the youngest reported in the literature to date.

Benign tumors of the palatine tonsil are less common than malignancies [2]. Differential diagnosis of a benign polypoid tonsillar lesion includes juvenile angiofibroma, fibroepithelial polyp, arteriovenous malformation, lymphangiectasia, and squamous papilloma [2, 3]. For a young child, such as our patient, the likelihood of a malignancy is unlikely, yet a lymphangiomatous polyp must be differentiated from a juvenile angiofibroma since the latter diagnosis warrants a more aggressive surgical resection and closer followup due to higher chances of recurrence [2].

The histopathologic features of a lymphangiomatous polyp that distinguish it from the other processes include dilated vascular channels containing luminal proteinaceous fluid and lymphocyte aggregation, which were present in the biopsied specimen in our case. When compared to other lymphatic malformations in the head and neck, the polypoid lesions differ in that they contain a greater degree of dense connective tissue and the lymphatic channels are not as prominently dilated.

The pathogenesis of tonsillar lymphangiomatous polyps remains unclear. Chronic inflammation and associated obstruction of lymphatic channels were previously suggested as a possible mechanism [2], but this is unlikely given that chronic tonsillitis occurs much more commonly than polypoid lymphangiomas [3]. As well, some patients with lymphangiomatous polyps are very young and do not have a history of pharyngotonsillitis, as seen in our patient. This indicates that another mechanism, such as an isolated hamartomatous proliferation, is most likely responsible for the etiology of these tumors [3, 10]. This is in keeping with the haphazard proliferation of elements that are normally found in the tonsillar tissue [3].

Lymphangiomatous polyps of the palatine tonsils have not been linked to other lymphatic lesions elsewhere in the head and neck region [3]. Yet, only one study reported imaging results to fully rule out other simultaneous head and neck anomalies [10]. Computed tomography scan was performed in the postoperative setting in our patient and no other head and neck tumors were identified. Thus, lymphangiomatous polypoid lesions do not seem to be associated with other lymphatic malformations as part of a generalized process, which has been reported in the lower gastrointestinal tract [7].

Regarding the management of the polypoid lesions, most authors suggest that a tonsillectomy is the curative procedure of choice [3, 10]. Our patient underwent a simple excisional biopsy of the lymphangiomatous mass and with one year followup, there were no signs of recurrence. An excision of the polypoid mass may be the only necessary procedure instead of a tonsillectomy.
